# Ocean acidification promotes broad transcriptomic responses in marine metazoans: a literature survey

**DOI:** 10.1186/s12983-020-0350-9

**Published:** 2020-02-17

**Authors:** Marie E. Strader, Juliet M. Wong, Gretchen E. Hofmann

**Affiliations:** 1 Department of Ecology, Evolution and Marine Biology, University of California Santa Barbara, Santa Barbara, CA 93106 USA; 2 Department of Biological Sciences, Auburn University, Auburn, AL 36849 USA; 3 Present address: Department of Biological Sciences, Florida International University, North Miami, FL 33181 USA

**Keywords:** Ocean acidification, Transcriptomics, Marine metazoans, Metabolism, Calcification, Gene expression

## Abstract

For nearly a decade, the metazoan-focused research community has explored the impacts of ocean acidification (OA) on marine animals, noting that changes in ocean chemistry can impact calcification, metabolism, acid-base regulation, stress response and behavior in organisms that hold high ecological and economic value. Because OA interacts with several key physiological processes in marine organisms, transcriptomics has become a widely-used method to characterize whole organism responses on a molecular level as well as inform mechanisms that explain changes in phenotypes observed in response to OA. In the past decade, there has been a notable rise in studies that examine transcriptomic responses to OA in marine metazoans, and here we attempt to summarize key findings across these studies. We find that organisms vary dramatically in their transcriptomic responses to pH although common patterns are often observed, including shifts in acid-base ion regulation, metabolic processes, calcification and stress response mechanisms. We also see a rise in transcriptomic studies examining organismal response to OA in a multi-stressor context, often reporting synergistic effects of OA and temperature. In addition, there is an increase in studies that use transcriptomics to examine the evolutionary potential of organisms to adapt to OA conditions in the future through population and transgenerational experiments. Overall, the literature reveals complex organismal responses to OA, in which some organisms will face more dramatic consequences than others. This will have wide-reaching impacts on ocean communities and ecosystems as a whole.

## Background

In 1999, coral biologists first raised concerns about how changes in the saturation state of seawater might impact calcification in this foundation taxon [1]. Other marine biologists followed suit with studies reviewing impacts on plankton [2] with the first studies on other marine metazoans appearing in 2004 [3]. Shortly thereafter, chemical oceanographers and modelers described the process of ocean acidification (OA) and provided predictions regarding the rate of change in ocean chemistry; this has framed the timing on the impacts of OA on marine biota [4–6]. Over the last decade, the OA research community has made significant progress identifying vulnerabilities in our food systems and ecosystems using a variety of techniques ranging from marine materials methods to genomics. While some species are robust to changes associated with OA (i.e., under-saturated conditions), others are highly sensitive, which will have far reaching implications on ocean systems. In natural systems that mimic OA, there are significant reductions in biodiversity and functional richness along CO_2_ gradients [7]. Losses of key species will drive changes in food-web dynamics, habitat restructuring and reductions in marine resources that support human societies [8].

Accumulated past research has highlighted vulnerabilities in calcification-dependent metazoans, which are sensitive to changes in carbonate chemistry, noting that there is a great deal of variability that depends on taxa as well as evolutionary and environmental history [9, 10]. OA negatively impacts traits such as calcification, growth, reproduction and survival in some calcifying taxa, while traits remain neutral in others [9]. Other reviews have illuminated the effects of OA on ecosystem processes [11, 12], physiological responses of organisms [9, 13], important parts of food systems, including shellfish aquaculture [14–16] and energy transfer across trophic levels [17–19]. Here, we provide an overview of how the transcriptome, a sensitive physiological trait [20], shifts in response to OA in marine metazoans, and what these transcriptomic responses represent in the context of other traits. We highlight the general commonality in findings across studies, exceptions to these trends, and important factors worthy of additional consideration, such as life-history and co-occurring environmental stressors.

**Fig. 1:**
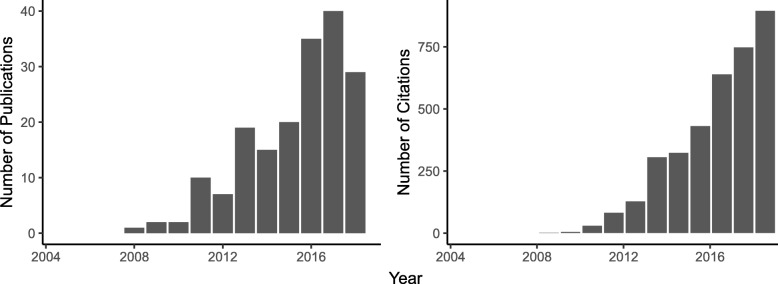
Web of Science search summarizing the number of papers and citations for papers with terms “ocean acidification” and “gene expression”

There has been a consistent increase in recently published works that incorporate transcriptomics in the study of organismal responses to OA: 76.1% of publications with ‘ocean acidification’ and ‘gene expression’ in the title or abstract have been published in the past 5 years (Fig. 1). Since changes in seawater chemistry impact physiological traits such as ion transport, metabolism and calcification, transcriptomics is an effective method for examining a molecular-level response that, when paired with phenotypic data, can elucidate mechanistic underpinnings for whole organism responses to OA. The increase in these studies emphasizes that transcriptome profiling is a robust and informative means by which to characterize how organisms are affected by environmental change.

## Overview of the studies

**Fig. 2:**
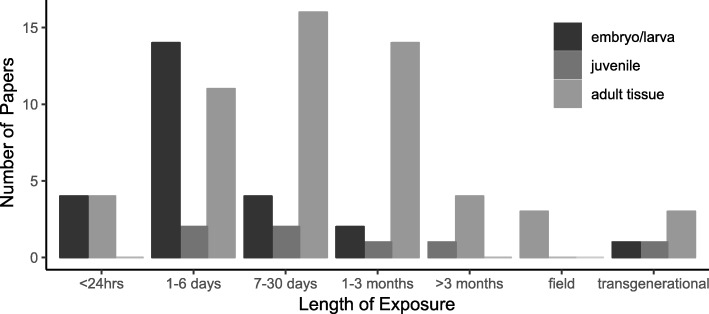
Exposure times in studies examining gene expression responses to ocean acidification across life-history stages. Field studies include those where samples were collected directly from field sites with different pH regimes. Transgenerational studies include those that examine progeny responses to pH stress after parental or grandparental conditioning in different pH environments. Gray scales represent the stage of the life-cycle sampled for gene expression analysis

Analysis of differential gene expression has been used to generate important observations about the response of marine metazoans to OA (Table 1). However, these studies can differ greatly in their approach, varying in the examined life-history stage, pH levels, exposure duration, and interactions in a multi-stressors framework (Fig. 2). For the most part, experiments have been conducted in the laboratory, although some studies have examined population-specific differences in gene expression across natural pH gradients in the field, illuminating how long term adaptation or acclimatization has impacted the transcriptome [23, 44, 78, 79, 87]. In addition, some studies have investigated organismal responses to acidifying conditions across two or more generations, providing further insight into the extent of phenotypic plasticity in an OA context [55, 80, 88, 89]. In our review of the literature, we found core transcriptomic responses to OA, but diversity in the ability of organisms to tolerate different pH environments. Below we summarize the collective results of these studies, noting five areas where gene expression analysis has contributed to our understanding of the physiology of marine animals and their response to OA. These key observations are that organisms: (1) differentially regulate metabolic pathways, (2) modulate genes involved in calcification and skeletogenesis, (3) activate the cellular stress response, (4) regulate acid-base ion transport mechanisms, and (5) alter behaviors. In addition, we note that there are often discrepancies between organismal responses to OA across life-history stages, where planktonic larvae and juveniles may be more sensitive to OA. Further, we examine the impacts of OA in the context of co-occurring multi-stressors and the evolutionary potential of organisms to adapt to OA.

**Table 1:** Gene expression studies examining the response of marine metazoans to OA

Phylum	Taxon	Species	Life-Stage	References
Cnidaria	Coral	*Acropora millepora*	Larvae/Juvenile	[21, 22]
			Adult	[23–26]
		*Acropora aspera*	Adult	[27]
		*Acropora gemmifera*	Larvae	[28]
		*Balanophyllia europaea*	Adult	[29]
		*Balanophyllia elegans*	Adult	[30]
		*Desmophyllum dianthus*	Adult	[31]
		*Pocillopora damicornis*	Larvae	[32]
			Adult	[33]
		*Siderastrea siderea*	Adult	[34]
		*Stylophora pistillata*	Adult	[29, 35]
			Cell culture	[36]
Annelida	Polychaete	*Platynereis dumerilii*	Adult	[37, 38]
		*Platynereis cfr massiliensis*	Adult	[37]
Mollusca	Abalone	*Haliotis rufescens*	Larvae	[39]
	Clam	*Ruditapes philippinarum*	Adult	[40]
		*Sinonovacula constricta*	Adult	[41]
	Mussel	*Mytilus californianus*	Larvae	[42]
		*Mytilus coruscus*	Adult	[43]
		*Mytilus edulis*	Adult	[44, 45]
	Oyster	*Crassostrea gigas*	Larvae	[46]
			Adult	[47, 48]
		*Crassostrea virginica*	Adult	[49]
		*Pinctada fucata*	Adult	[50–54]
		*Saccostrea glomerata*	Adult	[55–57]
	Pteropod	*Clio pyramidata*	Adults	[58]
		*Clione limacina*	Adults	[59]
		*Heliconoides inflatus*	Adults	[60]
		*Limacina helicina*	Adults	[61]
		*Limacina helicina antarctica*	Juveniles	[62]
		*Limacina retroversa*	Adults	[63]
	Scallop	*Pecten maximus*	Adults	[44]
	Snail	*Crepidula fornicata*	Larvae	[64]
Arthropoda	Copepod	*Acartia tonsa*	Adults	[65]
		*Calanus glacialis*	Larvae	[66]
		*Pseudocalanus acuspes*	Adults	[67]
	Crab	*Carcinus maenas*	Adults	[68, 69]
		*Hyas araneus*	Larvae	[70]
			Adults	[71]
Echinodermata	Brittle star	*Amphiura filiformis*	Adults	[72]
	Sea urchin	*Hemicentrotus pulcherrimus*	Embryos/Larvae	[73]
		*Lytechinus pictus*	Larvae	[74]
		*Mesocentrotus franciscanus*	Larvae	[75]
		*Paracentrotus lividus*	Embryos/Larvae	[76]
		*Strongylocentrotus droebachiensis*	Larvae	[77]
		*Strongylocentrotus purpuratus*	Embryos/Larvae	[78–86]
			Adults	[87]
Chordata	Fish	*Acanthochromis polyacanthus*	Juveniles	[88, 89]
		*Dicentrarchus labrax*	Larvae	[90]
		*Gadus morhua*	Adults	[91, 92]
		*Oryzias latipes*	Embryos/Hatchlings/Adults	[93]
		*Pagothenia borchgrevinki*	Adults	[94]
		*Sciaenops ocellatus*	Adults	[95]
		*Sebastes caurinus*	Juveniles	[96]
		*Sebastes mystinus*	Juveniles	[96]
		*Trematomus bernacchii*	Adults	[97]

### Observation 1: Organisms alter metabolic processes under exposure to low pH

As CO_2_ is absorbed into surface oceans, excess CO_2_ diffuses into extra- and intra-cellular compartments of marine organisms. Metabolic depression is a mechanism by which organisms conserve energy while cells actively work to decrease the influx in H^+^ ions through ion exchange processes [13]. Metabolic depression is typically an acute response since chronic reductions in metabolic processes can become lethal. While studies have investigated metabolic rates and ATP production in response to pH [98], gene expression has become another tool to assess the potential for metabolic depression, especially to elucidate mechanisms of this response (Table 2). Gene expression studies reporting metabolic depression in marine metazoans include adult blue mussels [45], adult pearl oysters [50, 51], purple sea urchin larvae [81, 82], temperate brittle stars [72], clams [41], Arctic copepods [66], developing Medaka fish [93], marine polychaetes [37] and reef-building corals [21, 24–26]. Despite being commonly observed across gene expression studies in response to low pH, the means by which metabolic depression is characterized through gene expression data varies by study and taxon.

**Table 2:** Impacts of high *p*CO_2_ on gene expression and oxygen consumption in marine invertebrate taxa. ‘Metabolic genes’ is a broad term that encompasses any metabolic processes as defined independently in each study. Arrows denote the direction of the response. In instances with both arrows, this reflects either overall differential expression (genes being both up and down regulated), or conflicting results across studies or life-history stages. Dashes indicate a non-significant response. It should be noted that oxygen consumption and gene expression measurements for a particular species may be confined to separate independent studies. Exposure duration and range of *p*CO_2_ tested are inclusive to all studies on noted species. In studies where *p*CO_2_ was not calculated (or only calculated in some studies), pH values are given. Hpf = hour post fertilization, dpf = days post fertilization, d = days, h = hour, wk = weeks, mo = months, field = organisms sampled from variable habitats in the field, generation = exposure carried out across multiple generations

Metabolism						
Phylum	Species	Metabolic genes in response to high *p*CO_2_	Oxygen consumption measured / response in high *p*CO_2_	Exposure duration	Range of *p*CO_2_ tested (μatm)	References
Cnidaria	*Acropora aspera*	↑ or ↓	N	14 d	142–827	[27]
	*Acropora millepora*	↓ or –	N	1 h - Field	325–1638	[21–26]
	*Balanophyllia elegans*	↑ or ↓	Y / - or ↓	29 d	785–2367	[30]
	*Desmophyllum dianthus*	↑	Y / ↑	8 mo	460–997	[31]
	*Pocillopora damicornis*	↑ or -	Y / ↓	24 h − 1.5 mo	417–3879	[32, 33, 99, 100]
	*Siderastrea siderea*	↑	N	95 d	280–2800	[34]
Mollusca	*Clio pyramidata*	↓ or -	Y/ -	10 h	280–800	[58]
	*Crassostrea gigas*	↓ or -	Y/ -	18hpf-3mo	449–1515	[46, 48]
	*Crassostrea virginica*	↑ or ↓	N	Field	pH:7.2–8.0	[49]
	*Crepidula fornicata*	↓	N	12 d	504–1480	[64]
	*Heliconoides inflata*	↓	Y/ -	3 d	382–720	[60]
	*Limacina helicina antarctica*	↓	Y/ ↑	1–21 d	215–961	[62, 101]
	*Limacina retroversa*	↑ or ↓	Y/ ↑	1–21 d	464–1177	[63]
	*Mytilus californianus*	–	N	63 hpf	345–1411	[42]
	*Mytilus edulis*	↓	Y / ↑	2d-2 mo	385–4000	[45, 102, 103]
	*Pinctada fucata*	↓	Y / -	0.5 h-2 mo	pH:7.4–8.1	[50, 51, 53, 104]
	*Saccostrea glomerata*	↑ or ↓	Y / ↑	1 wk. −5 wks	380–1329	[55–57, 105]
	*Sinonovacula constricta*	↑	Y / ↓	1 wk	549–3064	[41]
Annelida	*Platynereis spp.*	↑ or –	Y / ↑	5 d – 7 d	478–6534	[37, 106]
Arthropoda	*Calanus glacialis*	↓	Y / -	35–38 d	320–1700	[66, 107]
	*Hyas araneus*	↑ or ↓	Y / - or ↓	9d – 10wks	420–3300	[70, 71]
	*Pseudocalanus acuspes*	↑ or ↓	Y / ↑	generations	400–1550	[67, 108]
Echinodermata	*Amphiura filiformis*	↓	Y / ↓	4 wks	492–6399	[72]
	*Lytechinus pictus*	↓	N	142 hpf	280–970	[74]
	*Paracentrotus lividus*	–	N	3 d	397–6590	[76]
	*Strongylocentrotus droebachiensis*	↓	N	5.4 dpf – 16 mo	400–1200	[77, 109]
	*Strongylocentrotus purpuratus*	↑ or ↓	Y/ ↑, − or ↓	40 hpf – 21 dpf	380–9556	[74, 81, 82, 84, 85, 110]

One line of evidence that predicts metabolic depression is changes in transcripts that modulate the production of ATP. This has been observed in both calcifying and non-calcifying marine taxa. In adult mantel tissue of blue mussels, *Mytilus edulis*, a downregulation of 2 subunits of ATP-synthase occurs under 1120 and 2400 μatm but not at 4000 μatm [45]. This is in line with results found in purple sea urchin, *Strongylocentrotus purpuratus*, larvae; genes associated with the production of ATP and the tricarboxylic acid cycle (TCA), are downregulated in response to moderate *p*CO_2_ (540 ppm) [81]. Follow-up studies suggest that rather than a decreased production of ATP in response to OA, *S. purpuatus* larvae reallocate the total ATP produced; more ATP goes toward protein synthesis and ion transport when exposed to high *p*CO_2_, leaving less energy to maintain other cellular functions [83]. However, larvae from populations of *S. purpuratus* experiencing frequent low pH episodes in nature upregulate metabolic processes, including genes in the TCA, suggesting these populations have adapted by constitutively expressing genes that enable higher ATP production [78]. These studies emphasize that a core response of marine metazoans to decreases in pH involves a reallocation and/or changes in production of ATP, often as a trade-off in maintaining ion homeostasis, calcification and control of internal pH levels. Modulation of ATP-producing enzymes has also been reported in non-calcifying marine organisms in response to low pH, including Arctic copepods [66] and within brains of coral reef fish [89].

Another commonly reported gene expression signature that predicts metabolic depression is changes in genes involved in mitochondrial and oxidative metabolism. This is particularly evident in some species of reef-building corals, whose metabolism is complicated by their obligate symbiosis with algae in the family Symbiodiniaceae. Adult *Acropora millepora* show signatures of metabolic depression after *p*CO_2_ stress, although results are varied. Exposure for 14 days to a *p*CO_2_ up to 1638 μatm showed no regulation of metabolic candidate genes [26], yet adult exposure to longer periods of *p*CO_2_ stress, (i.e., 28 days at 1010–1350 μatm [24], 35 days at 997 μatm [25] and 37 days at 886 μatm [26]) elicited a response indicative of metabolic depression, including genes involved in the TCA cycle, ATP and NADPH production, oxidative metabolism and the mitochondrion electron transport chain. Acute *p*CO_2_ exposure of early juveniles of *A. millepora* decreased expression of mitochondrion and oxidative metabolism genes, but only in the highest *p*CO_2_ treatments (1000 ppm) [21], a result not found when juveniles were exposed to high *p*CO_2_ stress immediately following fertilization [22]. This suggests that despite metabolic depression early in exposure, early life-history stages of corals have a remarkable ability to acclimate to higher *p*CO_2_ levels over more prolonged periods of time, while adults only employ signs of metabolic depression under longer term, high *p*CO_2_ exposure. This observation does not seem to hold for all early life-history stages of corals. In *Pocillopora damicornis* larvae from French Polynesia, there was no observable change in expression of metabolism-related genes after an acute 24 h exposure to high *p*CO_2_ (~1030 μatm) [33]. Overall, reef-building corals vary in how they regulate their metabolism in response to elevated *p*CO_2_ even during early life-history stages, providing evidence that early stages might be more robust to environmental variability than previously predicted.

Additional studies on reef-building corals suggest increases in metabolic processes under high *p*CO_2_ conditions. In another acroporid, *Acropora aspera*, Ogawa et al. [27] found an upregulation of three candidate metabolism genes (i.e., GAPDH, Glycogen phosphorylase and Glycogen synthase) when simultaneously exposed to the maximum temperature of the experiment (4 days) and a downregulation after temperature was returned to a consistent + 2 °C heat stress [27], an experimental design aimed to simulate a bleaching event with concurrent elevated *p*CO_2_ (~ 800 μatm). Experiments exposing adult *P. damicornis*, which is likely a species complex, to 2180 μatm *p*CO_2_ for 3 weeks also found an upregulation of functional categories associated with metabolism, particularly oxidative phosphorylation, glycolysis and lipid and protein metabolic processes [32]. A highly stress-tolerant Caribbean coral *Siderastrea siderea* also exhibited elevated expression of metabolism-related genes under high *p*CO_2_ (2553 μatm), including genes associated with oxidative metabolism related to the mitochondrion [34]. These increases in metabolic processes while experiencing high *p*CO_2_ levels suggest that maintaining calcification under extremely acidified conditions requires higher metabolic demands in these species; although it is key to note that the high *p*CO_2_ levels tested in [32, 34], 2180 μatm and 2553 μatm, respectively, are not likely to be ecologically relevant. This difference in response compared with other reef-building corals suggests that some species may have evolved divergent physiological strategies for mitigating exposure to high *p*CO_2_. These strategies likely reflect different mechanisms of maintaining homeostasis during high *p*CO_2_ events. It must be noted, however, that the magnitude of overall transcriptomic response to elevated *p*CO_2_ in reef-building corals is relatively low [21, 22, 26, 34] compared to other stressors, suggesting robust mechanisms to tolerate forecasted OA scenarios. In addition, mechanisms to mitigate excess H^+^ ions likely vary based on the length and the magnitude of high *p*CO_2_ exposure.

Organisms employ different mechanisms of metabolic responses based on length of exposure to stressors. Upregulation of genes involved in lipid metabolism is observed in many studies of long-term *p*CO_2_ stress response in a variety of taxa: reef-building corals [21, 23, 25, 32], pteropods [62, 63], and Arctic copepods [66]. Concurrently, there are studies reporting increased lipid content in corals after exposure to increased *p*CO_2_ [111, 112]. Presumably, under long-term exposure to high *p*CO_2_, corals increase their production of lipids to compensate for reductions in skeletal growth. There is also differential regulation of fatty acid metabolism in *S. purpuratus* from populations experiencing variable pH regimes [78]. Increasing lipid and carbohydrate metabolism has been suggested as a mechanism to sustain calcification and ion homeostasis when experiencing long exposures of low pH, either episodically, as in upwelling scenarios, or chronically, as in volcanic CO_2_ vents. Long-term acclimation or adaptation to chronic pH stress likely involves modulation of lipid metabolism towards favoring lipid storage.

Although signatures of metabolic depression in response to pH are common, these patterns can often be complex and context dependent, often times only observed in response to extreme pH. Temperate brittle stars, *Amphiura filiformis*, live in burrows that vary in pH depending on depth. Laboratory experiments show an increase in resting metabolic rate under low pH levels naturally experienced in their burrows (pH 7.3), while animals exposed to more extreme pH values (7.0) showed a decrease in metabolic rate and a reduced capacity to regenerate broken arms [72]. These physiological measures are supported by gene expression, which show reduced expression of key metabolic genes (Lactate dehydrogenase and Glucose 6-phosphate dehydrogenase) [72].

Studies of transcriptomic responses to high *p*CO_2_/low pH exposure in shell-forming zooplankton show dramatic modulation of energetic processes. Both Arctic copepods and Antarctic pteropods show massive downregulation of much of the transcriptome after low pH exposure (1700 μatm and 902 μatm, respectively) [62, 66]. Decreased expression of mitochondrion and oxidative metabolism genes are also observed under high *p*CO_2_ (720 μatm) in Mediterranean pteropods, including suppressed expression of the entire protein synthesis machinery [60]. In Antarctic pteropods, similar patterns are observed under pH stress after 7 days, including the downregulation of metabolic processes and genes associated with protein modifications [62]. Additionally, Antarctic pteropods downregulate lipid metabolism genes under low pH levels, presumably indicating differential energetic needs under acidified conditions. In Arctic copepods, there is no observable physiological response to low pH [66], however, there is a striking transcriptomic response with a genome-wide downregulation of genes, including those associated with fatty acid/lipid metabolism and energy.

Interestingly, many studies elaborated above are often not paired with physiological measurements that would further support metabolic depression, while those that do often find mixed results (Table 2). In blue mussels, there is an increase in whole organism oxygen consumption under high *p*CO_2_ levels (1120 and 2400 μatm) [102] despite a down-regulation of ATP-synthase subunits in mantle tissue at the exact same *p*CO_2_ levels [45]. Taken together, these studies suggest whole organism measurements do not necessarily reflect energetic changes in different tissues in response to reduced pH, where in mantle tissue there is potentially a shift towards more anaerobic metabolism to support the demand for ATP [45]. In pearl oysters there is no significant effect of reduced pH (pH 7.7 and 7.4) on respiration rate [104] despite downregulation of metabolic pathways [50]. The authors instead report that maintaining acid-base equilibrium during *p*CO_2_ stress comes at a cost to biomineralization, since these animals show a reduction in net calcification rate [50]. In burrowing razor clams, *Sinonovacula constricta*, the depth at which clams burrow decreases with increasing *p*CO_2_ (549–3064 μatm) which is associated with decreases in oxygen consumption rates and Ca^2+^/Mg^2+^ ATPase activity [41]. Gene expression of TCA genes does not correspond with this pattern, however. Here, the authors conclude expression of the selected genes does not explain the differences in measured behavioral responses and physiological traits [41].

Studies that do report similar associations between downregulation of metabolic genes and physiological measurements include those in corals, brittle stars, snails and urchins. In *A. millepora*, photosynthetic capacity and light enhanced dark respiration are decreased under predicted ocean acidification scenarios, which is associated with downregulation of multiple metabolic pathways [25]. In brittle stars there is a significant reduction in resting metabolic rates at very low pH associated with deep burrows (7.0 pH) which pairs with downregulation of metabolism related genes in arm tissue at both 7.3 and 7.0 pH [72]. In *S. purpuratus* larvae there are only differences in oxygen consumption rates under high *p*CO_2_ (1100 μatm) when reared in warmer temperatures, an effect also seen in the regulation of histone transcripts, which the authors attribute to a depressed metabolism [82]. However, differences in larval oxygen consumption are evident as they develop into mature feeding larvae, eventually displaying increases in metabolic rate under elevated *p*CO_2_ (1318 μatm) [84]. Larval growth rate and metamorphosis at 6 h post hatching is delayed in the Atlantic slippersnail, *Crepidula fornicata*, under high *p*CO_2_ (1480 μatm), which coincides with an overall transcriptomic depression, including downregulation of multiple GO categories associated with metabolism and growth [64].

### Observation 2: Exposure to low pH conditions induces downregulation of calcification and skeletogenesis genes in some calcifying organisms, but not others

Assessing the complexity of calcification regulation under pH stress in marine metazoans is often difficult due to the fact that many genes involved in these processes are presumably taxon-specific, and further, are unlikely to be well annotated. However, some calcifying organisms have well characterized calcification-related gene families and pathways, including echinoderms and mollusks. Calcification in echinoderms begins very early in development, in the planktonic late gastrula stage, where fully developed skeletal rods are observed in early pluteus larval stages. High *p*CO_2_ reduces overall body size, growth rate and skeletal development in early life-history stages of echinoderms [74, 77, 82, 84, 113], although adult pre-conditioning can have effects on larval responses to elevated *p*CO_2_ [109, 114]. Studies across four different species of sea urchins find decreases in expression of genes critical to calcification and skeletogenesis in early life-history stages, particularly those involved in binding and sequestering Ca^2+^ ions, including *Msp130* [73, 74, 81, 109]. Across a developmental time-series of embryological and larval development in *S. purpuratus*, spicule matrix proteins were significantly downregulated under high *p*CO_2_ [84]. However, studies in urchins have revealed that the means by which calcification is regulated in response to *p*CO_2_ is complex and gene expression results have been mixed. When late gastrulae are exposed to variable *p*CO_2_ stress (800–1200 μatm) there is differential expression of many genes involved in skeletogenesis, while later stage larvae shows little to no differential expression of these genes [79]. However, when later stage echinoplutei are exposed to combinations of high temperature and *p*CO_2_ (1100 μatm), there is differential expression of cytoskeletal genes and a spicule matrix protein [82], suggesting that regulation of skeletogenesis is a complicated process that responds to combinations of environmental effects. This study ultimately revealed that reductions in skeletogenesis in *S. purpuratus* echinoplutei are not a consequence of a depressed metabolism, but rather that high *p*CO_2_ inhibits the ability of these organisms to calcify [82]. It is not likely that these results are due to developmental delay as echinoplutei are sampled using developmental landmarks, and not with skeletal development [113]. Carbonic anhydrases (CAs) are often implied to be involved in biomineralization although there is often no signature of their differential expression in response to increased *p*CO_2_, or there is differential expression of only a small proportion of annotated CAs [79, 81, 110], suggesting that the role of CAs in mediating shifts to biomineralization in urchins is complex.

Many shelled mollusks play significant roles in their respective ecosystems as suspension filter feeders, as well as hold high economic value as fisheries and aquaculture species [115]. Therefore, there has been significant attention in how OA will impact these species in regards to growth, reproduction and maintenance of populations. Adult molluscan shells are typically composed of aragonite or calcite, rely on shell organic matrices and begin formation in early planktonic stages of development. Studies on the impact of OA on calcification of marine mollusks find the majority of adult responses to low pH are neutral in regards to calcification rates, although most of the remaining studies show negative effects [115]. Despite overall neutral effects in adults, the majority of studies examining early life-history stages show widespread negative effects, particularly on larval size, survival, developmental rates, metamorphosis and shell normality [115]. Variation in responses across mollusks may be due to varying capacities to regulate pH at the site of calcification, dissimilarities in the mineralogy of shells, and differences of protective organic layers between taxa [115]. Furthermore, studies have shown that an organism’s shell minerology can change in response to OA [116, 117]. Impacts of decreased pH on shelled mollusks has been well reviewed already [115], so here we will briefly highlight gene expression studies that have enhanced this work.

Studies of gene expression responses in shelled marine mollusks generally support observations gained in physiological studies (Table 3). In Pacific oysters, *Crassostrea gigas*, exposure to high *p*CO_2_ (~1241 μatm) early in development results in a delay in shell formation and differential expression of extracellular matrix formation genes (i.e., shell matrix proteins), presumably involved in calcification [46]. In the planktonic mollusk, *Heliconoides inflatus,* all annotated calcification genes were significantly upregulated under mid-range *p*CO_2_ (617-720 μatm) including matrix proteins, metaloproteases, c-type lectins and mucins [60]. Reductions in net calcification rate in adult pearl oysters, *Pinctada fucata*, correspond to differential expression of calcium binding proteins, but not shell matrix proteins [50, 51]. In mantle tissue of blue mussels, there are decreases in expression of chitinase and calcium binding proteins at increasingly high *p*CO_2_ (385–4000 μatm), but not the majority of shell matrix proteins [45]. Therefore, it appears that high *p*CO_2_ impacts different aspects of shell production and growth across life-history stages, although levels of *p*CO_2_ across many of these studies can vary wildly. During shell formation in planktonic stages, expression of extracellular matrix genes, such as shell matrix proteins, become differentially regulated under high *p*CO_2_, while in adults, high *p*CO_2_ induced differential expression of genes regulating ion transport and acid-base homeostasis, including calcium binding [50], likely to maintain calcification.

**Table 3:** Impacts of high *p*CO_2_ on gene expression and calcification in marine invertebrate taxa. ‘Calcification genes’ is a broad term that encompasses any calcification or biomineralization processes as defined independently in each study, which includes acid-base regulation in some studies and not in others. Arrows denote the direction of the response. In instances with both arrows, this reflects either overall differential expression (genes being both up and down regulated), or conflicting results across studies or life-history stages. Dashes indicate a non-significant response. In studies where *p*CO_2_ was not calculated (or only calculated in some studies), pH values are given. It should be noted that calcification and gene expression measurements for a particular species may be confined to separate independent studies

Calcification						
Phylum	Species	Calcification genes in response to high *p*CO_2_	Calcification measured / response in high *p*CO_2_	Method	Range of *p*CO_2_ tested (μatm)	Refs
Cnidaria	*Acropora gemmifera*	↓	Y / ↓	SEM	389–1214	[28]
	*Acropora millepora*	↑ or ↓	Y / ↓	Change in weight	325–1638	[21–26]
	*Balanophyllia elegans*	↑ or -	N	–	785–2367	[30]
	*Desmophyllum dianthus*	↑	Y / -	Change in weight	460–997	[31]
	*Pocillopora damicornis*	↑ or -	Y / -	Total alkalinity anomaly	417–3879	[32, 33, 99, 100]
	*Siderastrea siderea*	–	Y / ↑ or ↓	Change in weight	280–2800	[34, 118]
	*Stylophora pistillata*	↑ or ↓	Y / ↑ or ↓	Sr incorporation, SEM	pH:7.20–8.1	[35, 36]
Mollusca	*Crassostrea gigas*	↓ or -	Y / ↓ or –	Cross polarized light microscopy/shell weight	449–1515	[46, 48]
	*Crepidula fornicata*	–	Y / ↓	Shell length/growth rate	504–1480	[64]
	*Heliconoides inflata*	↑	Y / ↓	^45^Ca uptake	382–720	[60]
	*Limacina helicina*	↑ or ↓	N	–	pH: 6.5–8.23	[61]
	*Limacina helicina antarctica*	↑ or ↓	N	–	318–902	[62]
	*Limacina retroversa*	↑	Y / ↑ or –	Calcein staining/image analysis	464–1177	[63]
	*Mytilus californianus*	–	Y / ↓	Larval shell length	345–1411	[42]
	*Mytilus edulis*	↓	Y / ↓	Shell morphology	385–4000	[45, 102, 103]
	*Pinctada fucata*	↑ or ↓	Y / ↓	Shell length, weight, hardness, calcium content, SEM, net calcification rate,	pH:7.4–8.1	[50–52, 54]
	*Saccostrea glomerata*	↑ or ↓	Y / ↓	Shell length	380–1329	[55–57, 105]
Echinodermata	*Lytechinus pictus*	↓	Y / ↓	Skeletal morphology	280–970	[74]
	*Paracentrotus lividus*	↑	Y / ↓	Skeletal morphology/^45^Ca uptake	397–6590	[76]
	*Strongylocentrotus droebachiensis*	–	Y / ↓	Body size	418–1145	[77]
	*Strongylocentrotus purpuratus*	↑ or ↓	Y / ↓	Skeletal morphology /body size/ Calcein pulse-chase experiments	380–9556	[81, 82, 85, 110, 119, 120]

The ability of reef-building corals to produce three-dimensional skeletal structures drives the capacity for corals to provide resources to people as well as ecological goods and services [121]. OA threatens calcification and skeletal formation making it essential to understand the magnitude at which projected OA conditions will impact calcification in reef-building corals. A meta-analysis projected that coral calcification will decline ~ 22% by the end of the century [122], although changes in calcification vary greatly between studies and species of coral, suggesting a scenario that will promote winners and losers. Therefore, recent studies have aimed to better understand mechanisms of calcification in reef-building corals in order to predict how changes in ocean pH will drive changes in the ecosystem services reef-building coral provide [123, 124]. For example, *Porites spp.* exhibit a decrease in skeletal density but not linear extension under future OA scenarios [124]. Many studies investigating gene expression responses to predicted OA scenarios have generally been complex across studies, life-history stages and species. Studies in *A. millepora*, have been performed across multiple contexts. When early recruits of *A. millepora* are exposed to high *p*CO_2_ (1000 ppm) during the onset of calcification, they exhibit both increased and decreased expression of many skeletal organic matrix proteins, known for their roles in CaCO_3_ deposition, and decreased expression of multiple CAs [21]. However, this response only appears under acute exposure to *p*CO_2_ stress (i.e., 3 days) as differential expression of these calcification-associated genes is not observed when juveniles are exposed directly after fertilization [22]. This highlights that early life-history stages of *A. millepora* may possess the capacity to acclimate to high *p*CO_2_ levels regarding their calcification ability. In adults of *A. millepora*, the results become more mixed. There was almost no differential regulation of extracellular matrix transcripts presumably involved in calcification after 28 days (at 1010–1350 μatm), which is also found at the phenotype level as no change in growth or calcification was observed [24]. However, a follow up study with a longer exposure (5 weeks at 997 μatm) found complex patterns of gene expression in which calcium channels and transporters were upregulated under pH stress while bicarbonate transporters and CAs were downregulated [25]. Another study, which used adults of *A. millepora* exposed to elevated *p*CO_2_ (up to 1638 μatm) for 14 and 37 days, found no differential expression of candidate calcification genes including CAs, galaxins and a bicarbonate transporter [26]. However, an early qPCR study found differential expression of CAs under temperature and *p*CO_2_ (827 μatm) combined stress, modeled as a natural bleaching experiment, where temperature was manipulated through time in *A. aspera* [27]. In addition, *A. millepora* from volcanic CO_2_ seep environments experiencing chronic moderate to high *p*CO_2_ (624-998 μatm) did not exhibit changes in calcification-related genes [23], suggesting calcification processes in corals are robust to changes in pH. However, reduced levels of net calcification in *A. millepora* were found from the same volcanic sites, suggesting corals invest more energy in generating tissue biomass rather than skeletal growth when under acidified conditions [23, 111]. An opposite pattern was observed in temperate corals exposed to OA conditions (997 μatm) for 8 months, in which there was no observed reduction in calcification or changes in respiration, yet there was an increased expression of enzymes presumed to play a role in skeletogenesis [31].

Exposure to low pH, under-saturated conditions shows complex yet overall minimal impacts on calcification genes in *A. millepora*, however, there is variation in responses across coral taxa. In *Pocillopora damicornis* experiencing extreme *p*CO_2_ (2180–3876 μatm), adults upregulated genes involved in calcification including bone morphogenic proteins (BMPs), CAs and galaxins after a 3-week low pH exposure [32]. In *Siderastrea siderea*, exposure to extreme *p*CO_2_ (2553 μatm) results in a slight reduction of calcification rate comparable to rates observed at pre-industrial *p*CO_2_ levels [118]. Gene expression associated with this effect includes differential expression of ion transport as opposed to calcification-associated genes, although this effect was much smaller than effects of temperature [34]. The combination of these studies suggests that these reef-building corals are generally able to mitigate local changes in *p*CO_2_ by regulating the pH at the site of calcification using physiological mechanisms not associated with altering calcification directly. Interestingly, in an extreme experiment where *Oculina patagonica* and *Madracis pharensis* were exposed to pH levels of 7.3 and 7.6 for 12 months, polyps were found to completely dissolve their skeleton and disassociate from the colony [125]. When transferred back to ambient conditions, the soft bodied polyps re-calcified and began to reform colonies [125]. Although not necessarily ecologically relevant, this suggests the extreme capacity for plasticity in these coral species exposed to low pH conditions and suggests that coral have established mechanisms that trade-off skeletogenesis and calcification processes in favor of increased energy reserves and body size [125].

Finally, there is sometimes a disconnect between phenotypic measurements of calcification/growth and expression of genes presumed to be regulating these phenotypes (Table 3). In addition, methods in which to quantify changes in calcification vary substantially by taxon, making interpretations across studies complex (Table 3). Despite this, many studies find strong ties between calcification and gene expression signatures in organismal responses to OA, often reporting differential expression of calcium channels and transporters, bicarbonate transporters, CAs and skeletal organic matrix proteins, although this process is likely complex and regulated by numerous factors. Differential regulation of many of these proteins, along with modifications in ion transport mechanisms, can enable organisms to cope with OA by regulating internal pH.

### Observation 3: High pCO_2_ exposure often induces the cellular stress response

In marine metazoans, the cellular stress response (CSR) is a well characterized short term means of physiological tolerance to abiotic stress, but can also induce apoptosis, in which damaged cells are removed [126, 127]. Here, the heat shock response, the rapid up-regulation of molecular chaperones (heat shock proteins – HSPs), has been a key finding in many transcriptomic studies done in an OA context. Notably, the impact of OA has been investigated intensely in aquaculture species. The Sydney rock oyster, ﻿*Saccostrea glomerata*, has experienced decades of artificial selection for growth and disease resistance, which interestingly also corresponds to resilience against low pH [105, 128, 129]. This resilient family of oysters shows increases in resting metabolic rates [105] and in genes associated with the CSR and immune system, such as HSPs, antioxidant enzymes glutathione S-transferases and C1q domain proteins under ambient conditions [55, 56]. These enzymes are then downregulated under transgenerational moderate *p*CO_2_ (625 μatm) when compared to a control family [55, 56]. This suggests that constitutive expression of CSR genes may mitigate potential cell damage from reduced pH through the maintenance of cellular homeostasis. In another oyster species, *C. gigas*, planktonic stages of a population from China show a reduction in protein levels of HSPs under high *p*CO_2_ (~1800 μatm) [130], while a population from the Pacific coast of the United States, exposed to a slightly lower *p*CO_2_ level (~1515 μatm) show no differential regulation of HSP transcripts [46], suggesting potentially different adaptation and acclimation strategies between populations. Antarctic pteropods also differentially regulate genes associated with the CSR at high *p*CO_2_ (902 μatm), including upregulation of HSP70 with decreases in other CSR components [62], while arctic copepods display downregulation of numerous molecular chaperones and genes associated with DNA repair and protein degradation [66], suggesting the CSR exhibits a complex response to low pH. In *S. purpuratus* there is evidence that genes involved in the CSR are modulated under high *p*CO_2_; rearing larvae in moderate (540 ppm) and high *p*CO_2_ (1020 ppm) resulted in a dose-dependent response involving downregulation of many genes associated with protein integrity, such as chaperones, and defense against oxidative stress [81]. Stress response genes were also downregulated in another urchin species, *Lytechinus pictus* under high *p*CO_2_ (970 ppm) [74]. Taken together, these studies imply that organisms experiencing high *p*CO_2_ could be more vulnerable to additional stressors because they may be compromised in their ability to mount an appropriate and robust CSR.

In contrast to planktonic stages of echinoderms, core HSP expression increased under both acute and prolonged exposure to high *p*CO_2_ (750 ppm) in *A. millepora* juveniles, suggesting a generic stress response [22]. In adult *A. millepora*, 28 day exposure to high and moderate *p*CO_2_ (700 and 1100 μatm) resulted in a downregulation of expression of HSPs [24], although there was an observed upregulation of genes associated with oxidative stress (catalase, FAD linked oxidase) and apoptosis (caspase, TRAFs, for example). A follow-up study on more genotypes of *A. millepora* and a 5 week pH stress exposure also found upregulation of oxidative stress related genes and genes associated with apoptosis, DNA repair and ubiquitination [25]. Finally, chronic pH stress corresponds to downregulation of HSPs in *A. millepora* [23] and upregulation of HSP70 in a cold water coral *Desmophyllum dianthus* [31]. Taken together, these studies suggest that in corals, acute exposure to low pH induced a stress response involving HSPs, but that expression pattern reverses after chronic exposure. Having reduced baseline levels of HSPs as a mechanism of tolerating low-term and chronic stress could prevent these organisms from mounting a robust stress response to additional stressors such as temperature, anoxia or eutrophication. Coral-specific responses to acidosis are likely complicated by the increase in reactive oxygen species (ROS) due to stress from their algal symbionts. In terms of reef-building corals and other obligate symbiotic marine organisms, it is critical to consider a holobiont perspective, as differences in gene expression changes in hosts under global stress scenarios could be reflective of variable interactions with algal symbionts and other associated microbiota. It is possible that differences in gene expression signatures across reef-building coral species and life-history stages could be due to differences in interactions between the host and stress response machinery of algal symbionts or other associated organisms.

### Observation 4: Ion transport and acid-base homeostasis is modulated in response to low pH

When marine metazoans are faced with influxes of H^+^ ions, membranes will strive to re-establish their acid-base equilibrium. This ion transport depends on key transporters including v-type H^+^ ATPases, Na^+^/H^+^- and Na^+^ dependent Cl^−^/HCO_3_^−^ transporters [13]. Marine fishes are thought to be more robust to increases in *p*CO_2_ than invertebrates due to their high capacity to maintain acid-base homeostasis, although there is variability in sensitivities across fish taxa [131]. Atlantic cod, *Gadus morhua*, exposed to 3 different *p*CO_2_ levels (550 μatm, 1200 μatm, 2200 μatm) across 2 temperatures (10 °C, 18 °C) for 4 weeks showed temperature-dependent responses in multiple ion transport proteins, often with dose-dependent responses across *p*CO_2_ levels in gill tissue [91], while temperature had a stronger impact on expression of these same ion transport proteins in intestines [92]. These studies are further substantiated by protein levels of the same transport proteins. There is no evidence of differential regulation of H^+^ transport genes in *Acanthochromis polyacanthus* juveniles after parental exposure to elevated *p*CO_2_ (754 μatm) although there is regulation of key circadian rhythm genes, which may tie into osmoregulation processes [88]. However, other fishes increase expression of acid-base homeostasis genes, indicative of higher sensitivity to elevated *p*CO_2._ Early life-history stages of medaka fish, *Oryzias latipes*, exhibit developmental delay and consistent upregulation of 2 key acid-base homeostasis genes in response to elevated *p*CO_2_ [93]. Upregulation of acid-base homeostasis genes is also evident after 7 day combined temperature and *p*CO_2_ (1000 μatm) exposure in *Trematomus bernacchii* [97] but not in *Pagothenia borchgrevinki* [94]. These transcriptomic studies reveal differences in response to elevated *p*CO_2_ across different fish species, similar to what is reported in physiological studies [131]. Although it should be noted that there are actually few studies that connect gene expression changes in ion transport with actual measurements of external pH and HCO_3_^−^ levels (Table 4). Overall in fishes, although there are strong mechanisms to regulate acid-base homeostasis, future OA conditions are likely to elicit stress responses in some fishes.

**Table 4:** Impacts of high *p*CO_2_ on gene expression and acid-base homeostasis in marine invertebrate taxa. ‘Acid-base homeostasis genes’ is a broad term that encompasses any gene involved in proton (H^+^) and bicarbonate (HCO_3_^−^) transport as defined independently in each study. Arrows denote the direction of the response. In instances with both arrows, this reflects either overall differential expression (genes being both up and down regulated), or conflicting results across studies. Dashes indicate a non-significant response. It should be noted that physiological and gene expression measurements for a particular species may be confined to separate independent studies

Acid-Base Homeostasis						
Phylum	Species	Acid-base homeostasis genes in response to high *p*CO_2_	External *p*CO_2_ / response in high *p*CO_2_	HCO_3_^−^ levels / response in high *p*CO_2_	Range of *p*CO_2_ tested (μatm)	Refs
Arthropoda	*Hyas araneus*	↑ or ↓	Y / ↑	Y / ↑	420–3300	[71]
	*Carcinus maenas*	–	Y / -	Y / -	40–440 Pa	[68, 69]
Chordata	*Acanthochromis polyacanthus*	–	Y / ↑	Y / ↑	414–1900	[88, 89, 132]
	*Gadus morhua*	↑ or ↓	N	Y / ↑	550–2200	[91, 92, 133]
	*Oryzias latipes*	↑	N	N	393–7081	[93]
	*Pagothenia borchgrevinki*	–	N	N	427–1053	[94]
	*Trematomus bernacchii*	↑	N	N	430–1000	[97]
Echinodermata	*Strongylocentrotus purpuratus*	↓ or -	Y / ↓ or -	N	380–9556	[81, 85, 110, 134]
	*Amphiura filiformis*	↓	Y / ↓ (pH)	Y / ↑	492–6399	[72]

Despite their presumed higher sensitivity to *p*CO_2_ than fish regarding acid-base homeostasis, marine invertebrates actually exhibit robust responses to elevated *p*CO_2_ as well. Early life-history stages of sea urchins regulated ion transport and acid-base homeostasis genes during development [85, 135, 136], but nevertheless, low pH exposure also impacts expression of some genes involved with ion transport, mainly those involved in Ca^2+^ regulation, as discussed in detail in Observation 2 [74, 79, 81]. There was little to no differential expression of genes involved in transmembrane movement of H^+^ ions when embryos and larvae were exposed to increased *p*CO_2_ [84], and across populations with different pH regimes [79] although there was significant downregulation of proton and Na^+^-dependent ion transporters in [74, 81] suggesting decreased capacity for ion transport under high *p*CO_2_. Measurements of internal midgut pH across various seawater pH levels showed stable gastric pH levels until seawater pH reached 7.22, at which time internal pH plummeted [85]. Overall, regulation of internal pH appears relatively stable in *S. purpuratus*, supporting the notion that these animals are able to internally modulate their cellular pH without adjusting their transcriptome [79, 81]. Acid-base homeostasis is altered more substantially in other echinoderms exposed to low pH. In brittle stars, extracellular pH in coelmic cavities and body fluid HCO_3_^−^ decreased and increased, respectively, when exposed to pH 7.6 and 7.2 for 16 days, which corresponds to downregulation of some key ion regulation genes [72]. In general in calcifying organisms, ion transport and acid-base homeostasis are heavily intertwined with biomineralization and calcification mechanisms, which was previously discussed in more detail (Observation 2).

Acid-base homeostasis is disrupted in chitinous marine invertebrates as well. In spider crabs, *Hyas araneus*, body hemolymph HCO_3_^−^ increased under intermediate (1120 μatm) and high (1960 μatm) *p*CO_2_ while hemolymph *p*CO_2_ also increased in a dose-dependent manner, suggesting an inability to compensate to seawater with elevated *p*CO_2_ [71]. Expression of v-type H^+^ ATPases and subunits of carbonic anhydrases were increased under elevated *p*CO_2_ [71], further substantiating the sensitivity of this species to OA conditions. In the green crab, *Carcinus maenas*, hemolymph *p*CO_2_ and HCO_3_^−^ levels did not change after 7 days at 324 Pa, although K+ and ammonia concentrations significantly increased [68]. Expected acid-base homeostasis genes were not differentially expressed in this species, although additional candidates genes were revealed by this study [69]. Reef-building corals also modulate ion transport genes in response to different pH levels. Ion transport genes were not differentially regulated in juvenile *A. millepora*, with the exception of upregulation of 2 T-type Calcium channels [21]. In adult *A. millepora*, exposure to *p*CO_2_ stress for 28 days resulted in differential regulation of membrane transporters [24]. This included upregulation of sodium and potassium transporters, ABC transporters and cell membrane receptors as well as a lipid transporter, suggesting elevated *p*CO_2_ levels may impact lipid configurations in cell membranes [24]. Under extreme *p*CO_2_, the coral *Siderastraea siderea* highly upregulates H^+^ ion transport and ATP synthesis genes, suggesting this species works to regulate pH at the site of calcification under extreme *p*CO_2_ [34]. Adults of *Pocillopora damicornis* also upregulate genes associated with ion transport and regulation, including Ca^2+^ and HCO_3_^−^ transporters. However, at extremely low pH values this pattern was reversed [32]. Overall, corals harbor robust mechanisms to maintain acid-base homeostasis under OA stress with complex effects on calcification processes as detailed above in Observation 2. These studies combined suggest that ion transport and acid-base homeostasis are mechanisms at the forefront of responding to low pH, and fine details of how these processes are regulated under varying pH regimes can be variable in regards to the magnitude of the stress, the life-history stage and the taxon.

### Observation 5: Neural functions and behavior are modulated by low pH through changes in ion transport mechanisms

Organismal responses to OA shape larger-scale ecological processes through altering animal behaviors, including settlement, predation, foraging and habitat use [137]. Transcriptomic studies investigating mechanisms of these altered behaviors in response to OA are restricted to a few systems. While there are several reviews on the impacts of OA on fish behavior [137–142], we will briefly highlight the studies that link gene expression and physiological responses. In a variety of marine fish, high *p*CO_2_ can dampen responses to alarm and predator cues, behaviors that have large impacts on survival [137, 143, 144]. Physiological studies on this phenomenon suggest that this behavioral response is due to changes in acid-base ion transport that work to prevent acidosis, with downstream impacts on GABA function in the brain [145]. Complex transgenerational experiments have been performed in spiny damselfish, *Acanthochromis polyacanthus*, to parse out how plasticity in this behavioral response may be propagated across generations and how OA impacts brain function on a transcriptomic level. Individual fish that are sensitive or tolerant to OA, measured by their behavioral responses to predator alarm cues, were crossed with fish of similar phenotypes, and the progeny was reared at either control or high *p*CO_2_ conditions [88]. Gene expression in the brains of these fish suggest that offspring from tolerant parents have flexibility in their ion regulation and can shift their physiology to avoid maladaptive responses to high *p*CO_2_. A follow-up study also finds differential expression of genes associated with behaviors, ion regulation and GABA pathways when fish were exposed to high *p*CO_2_ acutely and throughout development, whereas these signatures returned to baseline levels when parents were previously exposed to high *p*CO_2_ [89]. Differential gene expression signals of transgenerational plasticity in response to CO_2_ varied between different parental genotypes, suggesting individual fish have different tolerances and sensitivities to how their brains regulate ion homeostasis, prevent acidosis and regulate GABA pathways [89]. European sea bass also show impaired sensory function under high *p*CO_2_ conditions, which was associated with differential expression of glutamate sensory pathways and genes associated with synaptic plasticity [144]. Taken together, these findings suggest that fish lose sensory acuity in their environment under high *p*CO_2_ conditions, an outcome that would have strong implications on fitness in situ.

OA also potentially impacts behaviors or neurological traits in planktonic mollusks. In *Heliconoides inflatus* pteropods that experienced acute exposures to three different *p*CO_2_ conditions, 22% of the differentially expressed genes were involved in nervous system functioning, including many ligand-gated ion channels, GABAergic, cholinergic and glutamatergic [60]. In contrast, an opposite pattern was found in *Limacina helicina* pteropods, in which there was a decrease in expression of many neural-associated genes, although these pteropods experienced a longer exposure to high *p*CO_2_ conditions [62]. There was, however, similar patterns of upregulation in genes associated with acetylcholine transport. These results reveal that low pH stress has the potential to impact a variety of behaviors in pteropods, which should be investigated as they could have profound effects on their biology and function within their respective ecosystems.

### Observation 6: The transcriptomic response to low pH conditions vary by life-history stage

OA effects on organisms and their transcriptomes can vary by life history stage. In general, early developmental stages (EDSs) are believed to be the most vulnerable times during the life-history of most marine organisms [146–148], and may act as the bottleneck that determines whether a species will be successful in the future [148–150]. Life-history strategies vary greatly across marine metazoan taxa, and must be carefully considered when examining transcriptomes and determining susceptibilities to OA. For instance, marine larvae are often planktonic, but some species undergo direct development in which there are no true larval stages or metamorphoses [151, 152]. OA will differentially impact benthic versus pelagic EDSs because they experience disparate habitats and thus selection pressures. Larvae may be either calcifying or non-calcifying, and may be planktotrophic (i.e., feeding) or lecithotrophic (i.e., non-feeding), which may also impact their gene expression patterns and ability to cope with OA [103, 148, 149, 153]. Furthermore, the large expenditure of energy required for metamorphosis may place juvenile stages at increased risk to pH stress [109, 154] and negative impacts at earlier life stages may carry over into later stages, altering demography and distributions [155]. All of these factors are important considerations when designing and evaluating transcriptomic studies of EDSs in response to low pH.

Developmental staging is critical in transcriptomics on EDSs [156]. A detailed developmental transcriptome can be a useful means by which to identify the molecular profiles of different stages and identify genes that are important during major developmental landmarks. It is also worth noting that low pH conditions can lead to delayed early development [84, 157]. Thus it is vital to ensure the same developmental stages are compared regardless of different pH exposures. We recommend that EDSs are sampled based on observations of developmental progression rather than by a set number of hours post-fertilization. Despite these considerations, understanding the effects of low pH on early development remain difficult largely due to intraspecific differences caused by genetic variation, variances in maternal investment, and phenotypic plasticity [158]. However, transcriptomics has been successfully used to investigate how EDSs respond to OA.

In general, studies investigating the effects of OA on the EDSs of sea urchins have found that low pH impacts genes involved in skeletogenesis, cellular stress response, ion regulation and transport, apoptosis, spicule matrix proteins, and metabolism [73, 76, 79, 81, 82, 110, 159]. Runcie et al. [77] used a quantitative genetic breeding design to examine the role of genetic variation in larval tolerance of *S. droebachiensis* larvae to low pH, and found that changes in larval gene expression were more closely tied with differences of parentage than with differences of experimental pH treatments. In *S. purpuratus*, the exposure of adult female sea urchins to simulated upwelling conditions (i.e., high *p*CO_2_ in combination with low temperature) altered the transcriptomic response of gastrula stage embryos to high levels of *p*CO_2_ during early development [80]. These studies highlight that parental effects can greatly impact transcriptomic responses to OA in EDSs.

The transcriptomic response to OA has also been observed in fish EDSs. Fish can be more susceptible to low pH conditions during their early development [160], which may be due to the lack of fully developed organs that regulate ion exchange (e.g., gills, intestines, and kidneys) prior to reaching adulthood [93]. Transcriptomic analyses of the ricefish *Oryzias latipes* showed that embryos and hatchlings were more sensitive to OA than the adults, which the authors attribute to absence of fully developed ion regulatory epithelial tissues [93]. In contrast, larval fish of the European sea bass *Dicentrarchus labrax* exhibited no changes in gene expression across different *p*CO_2_ levels [90]. Even between two congeneric rockfish, Hamilton et al. [96] found that the transcriptomic response of juvenile rockfish to high *p*CO_2_ varied by species, indicating that fish sensitivity to OA during early development is largely species-specific. Lastly, parental effects can influence the gene expression patterns of fish during early development. For instance, in the damselfish *Acanthochromis polyacanthus*, the expression of genes related to glucose regulation and histone variants varied between offspring of adults that were behaviorally tolerant or sensitive to high *p*CO_2_ [88]. Furthermore, transgenerational exposure to high CO_2_ conditions altered offspring gene expression to baseline levels that were similar to those found in control fish raised under present-day CO_2_ conditions. Thus, the transcriptomic response of juvenile offspring to acidified conditions were shown to vary by both parental phenotype and by parental exposure to high *p*CO_2_ [88].

Unlike other systems, the early life-history stages of reef-building corals may be relatively resilient to OA when compared to their adult counterparts. This may be because coral larvae do not actively undergo calcification processes; the expression of genes related to biomineralization increases upon settlement [161]. For example, there were greater changes in gene expression of adult *P. damicornis* under high *p*CO_2_, particularly of genes related to calcification processes [32], than of larval *P. damicornis* subjected to acute high *p*CO_2_ exposures [33]. Specifically, Rivest et al. [33] found a stronger transcriptomic response in the algal symbionts of *P. damicornis* than in the larvae themselves. In other reef-building coral species, symbiosis is established horizontally, typically after metamorphosis and during early skeletal formation. Thus, one benefit of examining EDSs of horizontally transmitting corals is that their transcriptomic responses are not confounded by obligate algal symbiosis [21]. Acute exposure to low pH conditions impacted newly settled coral polyps by altering expression patterns of genes related to skeletal organic matrix proteins involved in calcium carbonate deposition processes [21]. However, given a longer exposure period, juvenile coral polyps appeared to acclimate to elevated *p*CO_2_ levels via an increase in expression of anti-apoptotic genes [22]. Overall, while EDSs are thought to be more vulnerable than adults, transcriptomic evidence supports that coral EDSs may be surprisingly robust to OA.

### Observation 7: OA interacts with other abiotic factors in a multi-stressor context

Changes in ocean pH can naturally vary in tandem with other environmental factors. For instance, upwelling brings waters characterized by a combination of low pH levels, low temperatures, and low oxygen concentrations [162, 163]. In systems dominated by benthic algae and macrophytes, biotic processes such as photosynthesis can result in high positive correlations between pH and oxygen [164, 165]. Natural variations in ocean conditions, all of which vary greatly by habitat and region [166, 167], will be further complicated by anthropogenic impacts, such as increasing absorption of atmospheric CO_2_ into the open ocean [167] or coastal nutrient enrichment [168]. As climate change continues, OA is not expected to occur in isolation; other environmental stressors are anticipated to intensify in the future as well [169–171]. Because factors such as ocean pH, temperature, salinity, and oxygen levels are predicted to change, often simultaneously, a multistressor approach may be required to accurately predict organismal responses to future marine environments [150, 172].

Studies that have used transcriptomics to investigate OA in a multistressor context have focused primarily on temperature as an additional stressor. O’Donnell et al. [75] subjected larvae of the red sea urchin *Mesocentrotus franciscanus* raised under different *p*CO_2_ levels to acute temperature treatments to determine how OA affected the thermal stress response. This study found that following a one-hour exposure to elevated temperatures, larvae raised under elevated *p*CO_2_ showed lower expression of the molecular chaperone HSP70. HSP70, which plays an important role in cellular defense, is controlled by a temperature sensitive promoter. Thus, a measured decrease in expression of HSP70 as a result of elevated *p*CO_2_ exposure suggests that larvae exposed to lower pH conditions may be more vulnerable to heat stress [75]. Other studies examined the transcriptomic response of organisms exposed to low pH and temperature stress at the same time. Simultaneous exposure to elevated *p*CO_2_ levels and temperatures has been shown to induce a transcriptomic stress response in coral and their symbionts [27, 29, 33], oysters [50–52, 56], crabs [71], sea urchins [82], and fishes [91, 94, 97]. These studies have examined temperature in the context of ocean warming, in which temperatures are elevated relative to average, ambient temperatures. While this is certainly pertinent given rising global temperatures, it is also worth noting that the intensity and duration of upwelling events are expected to increase in the future [173]. As such, it is also relevant to examine how combinations of high *p*CO_2_ with low temperatures, such as during upwelling events, will affect the transcriptome.

Other studies have investigated the combined effect of OA and chemical contamination on gene expression, particularly in bivalve species that inhabit coastal regions impacted by pollution [40, 47]. The negative immunosuppressive effect of cadmium exposure on the oyster *C. gigas* was intensified by simultaneous exposure to acidified conditions [47]. Specifically, the combined exposure of low pH and cadmium led to an increase in expression of genes involved in the Toll-like receptor (TLR) signaling pathway and immune factors, tumor necrosis factor (TNF) and integrin beta-1B. In the clam *Ruditapes philippinarum*, low pH affected the toxicity of pharmaceutical drugs, altering the expression of genes related to metabolism, neurotransmission, and the drug mode of action [40]. Overall, these studies show that OA can interact with contaminants, altering toxicity and organism sensitivity to different chemical pollutants.

In general, there are three major classifications of stressor interactions: (1) additive effects in which the combined effect is equal to the sum of the individual effects, (2) synergistic effects in which the combined effect is greater than the sum of the individual effects, and (3) antagonistic effects in which the combined effect is less than the sum of the individual effects [174, 175]. Most studies using transcriptomics to investigate multiple stressors including acidification have found evidence of synergistic effects [27, 29, 47, 49, 52, 94, 97], although there are several exceptions to this. Padilla-Gamino et al. [82] found that elevated *p*CO_2_ and temperature had an additive effect on *S. purpuratus* larvae, and concluded that ocean warming would not exacerbate the impaired skeletal growth caused by increased *p*CO_2_. In the Sydney rock oyster, *S. glomerata*, Goncalves et al. [56] found that the effects of *p*CO_2_ and temperature were neither additive nor synergistic, and were potentially antagonistic, in which temperature may have offset the effects of elevated *p*CO_2_. Adverse effects of OA on organism growth and calcification have been shown to be ameliorated by warming in some species [150, 168, 176, 177], although there is little evidence of this at the transcriptomic level. In contrast, Davies et al. [34] reported that the coral *S. siderea* was more negatively affected by high temperature than low pH, with transcriptomic patterns providing evidence of cellular shutdown under warming conditions while there was potential for acclimation to OA (i.e., an upregulation of genes related to ion transport).

While studies have examined the transcriptomic response of combined temperature or chemical pollutant stress with OA, environmental variables such as oxygen levels or salinity are largely absent from transcriptomic multi-stressor studies. Furthermore, nearly all studies are limited to two stressors. This is understandable, as manipulating more than two factors can lead to highly complex experimental design and difficult data interpretation. The challenge lies in choosing the appropriate combination of stressors that is ecologically relevant and accurately reflects what organisms are likely to experience in their current and future environments. Field experiments performed in situ allow for the full combination of environmental factors that organisms are experiencing in nature. One such study by Chapman and colleagues examined gene expression patterns in oysters across several environmental factors, including pH, temperature, salinity, dissolved oxygen, turbidity, chlorophyll a, ammonium levels, and metal and organic contaminants [49]. Changes in the transcriptome were primarily controlled by environmental pH and temperature, although salinity and dissolved oxygen also explained some patterns in the transcriptome. Combined low pH and high temperature stress decreased expression of genes involved in protein synthesis and cell growth, and increased expression of genes related to ATP production (i.e., mitochondrial oxidative phosphorylation.) Metal and organic contaminants, however, had a minimal impact of the observed gene expression patterns. Overall, this study examined how transcriptome patterns changed with respect to individual stressors as well as combinations of a multitude of stressors, providing important insight into how organisms respond to their highly variable and complex environments.

## Evolutionary potential to adapt to OA

Many early ocean acidification studies selected experimental pH levels based on IPCC (Intergovernmental Panel on Climate Change) projections for atmospheric concentrations of CO_2_ or average surface ocean pH levels predicted for the year 2100. However, as we learned more about in situ pH in coastal oceans (Hofmann et al. 2011), these pH levels were often not ecologically relevant for the range of marine ecosystems under study. Early OA studies often exposed organisms to high *p*CO_2_ levels that are likely ecologically unlikely, or did not include natural dynamics of *p*CO_2_ fluctuation, thus complicating the interpretation of organismal response to near future acidification projections. In addition, awareness started to grow regarding the influence of multiple stressors [178]. For instance, other anthropogenic effects (e.g., nutrient enrichment) contribute to decreases in pH, particularly near heavily populated coastal areas [179, 180]. The culmination of these anthropogenic impacts act in combination with natural variations in ocean pH, resulting in pH conditions that can vary dramatically by habitat and location [166, 167]. In order to understand adaptive potential, it is critical to frame studies within an organism’s natural environment, especially through time. Although these early studies were informative, particularly in regards to mechanisms enabling regulation of internal pH and calcification, we sometimes missed the opportunity to explore plasticity, and the potential for organisms to adapt to future ocean pH values. Many calcifying organisms have been exposed to fluctuations in ocean pH throughout their evolutionary history, however, it is yet to be determined if organisms can evolve rapidly enough to combat the quick pace of OA since the Industrial Revolution. Strategies to investigate adaptive potential to OA include 1) comparative population studies that examine natural variation in stress response phenotypes, and the genetic basis behind this variation, across landscapes that vary in pH, 2) studies that examine organismal responses to pre-industrial ocean pH as a treatment in short term response studies, 3) studies that examine adaptive responses to pH across multiple generations and 4) studies that incorporate environmental sensor data to inform experimental conditions based on the natural variability within the environment.

Comparative population studies have been performed in *S. purpuratus* inhabiting the California Current System, where variation in exposure to pH is likely to drive local adaptation in sensitivity to OA [78, 87]. In a common garden approach that investigated natural variation in gene expression of larvae from populations experiencing varying frequencies of low pH episodes, along with differences in temperature and oxygen, Evans et al. [78] found that larvae from populations experiencing more frequent low pH episodes upregulated key metabolic processes normally downregulated under pH stress. Therefore, it is likely that when *S. purpuratus* populations are chronically exposed to higher frequencies of low pH, as in the northern populations, they have adapted by constitutively expressing genes that enable higher ATP production, a pattern also found in a comparative population study in eastern oysters, *C. virginica* [49]. In another comparative study of *S. purpuratus* populations, Pespeni et al. [181] used a common garden approach and found that genes involved in carbohydrate and lipid metabolism were under selection, providing further evidence that chronic responses to low pH involve generating higher energy stores in the form of carbohydrates, lipids and ATP. Additional evidence for local adaptation to variable pH regimes is evident when examining differences in biomineralization genes; adult urchins from southern populations overexpress biomineralization genes when exposed to lower pH regimes for 3 years [87].

CO_2_ seep environments provide a window into community and organismal responses to chronically high *p*CO_2_ and give opportunities to observe acclimatization and adaptation in a natural environment [182, 183]. Volcanic CO_2_ seep sites in Papua New Guinea (PNG) and the Mediterranean exhibit differences in community composition and variation in taxon representation, suggesting that some species are intolerant of these extreme environments while some have found mechanisms of acclimation or adaptation [7, 184]. In addition, there are several seep environments in each region, providing site replication to further substantiate observations in these unique environments. While physiological studies of organisms inhabiting CO_2_ seeps have begun to unravel the effects of chronic *p*CO_2_ stress [111, 185], only one study to date has investigated differences in the transcriptome between individuals living within the seep environments and individuals within sites directly nearby the seeps but with ambient conditions [23], although additional proteomic investigations have also been performed [186]. In the transcriptomic study, gene expression of *A. millepora* individuals and their algal symbionts from two CO_2_ seeps and nearby control sites revealed a core expression response associated with individuals living in those extreme environments [23]. This response was associated with differential expression of very few genes, but included downregulation of molecular chaperones and alterations in fatty-acid lipid metabolism from individuals in the seep sites. This study highlights that chronic low pH environments generate very different gene expression responses than acute exposures typical of laboratory experiments.

Other studies in reef-building corals have examined organismal responses to pre-industrial ocean pH levels. ﻿*Siderastrea siderea* shows reductions in calcification rates at pre-industrial *p*CO_2_ levels (324  μatm) that mirror those at extreme *p*CO_2_ (>2500 μatm), however, there was minimal changes in the transcriptome associated with the preindustrial levels [34]. In *A. millepora*, increased rates of photosynthesis were observed when corals were exposed to pre-industrial *p*CO_2_ levels [25]. In addition, there appears to be a complex regulation of metabolic processes when corals were exposed to 5 weeks of pre-industrial *p*CO_2_ levels, such as the upregulation of cell cycle-related genes and glycolysis with a downregulation of Calvin cycle genes [25]. These results together suggest that *S. siderea* may be better adapted to pre-industrial conditions and are still in the process of adapting to current *p*CO_2_ levels. If there does exist this lag in evolutionary responses to the environment, it could be further evidence that although corals can evolve and adapt to changes in *p*CO_2_, the rate at which *p*CO_2_ levels in the ocean are increasing may be faster than the rate at which these corals can respond.

In addition to pH levels, exposure frequency and duration are important considerations in transcriptomic studies investigating adaptive capacity to OA. This includes incorporating fluctuating and variable pH conditions that occur in the natural environment into experimental designs. There is a great degree of natural variability in *p*CO_2_ across marine ecosystems [187], something the research community has documented at high spatiotemporal resolution through use of autonomous pH sensors. Such considerations are particularly relevant when reflecting upon when during an organism’s life cycle exposure to low pH occurs as well as the length of a species’ generation time. Many transcriptomic studies in marine systems that have examined differences in exposure frequency and duration have been rooted in toxicology [156, 188, 189]. Here, however, we briefly highlight a few examples of OA-focused transcriptomic studies that have tailored their experimental design to match the ecology of their study system. Transcriptomic studies that fail to frame their investigations within an ecologically relevant context risk inaccurately predicting how marine organisms respond to low pH in nature.

Evans et al. [79] measured the naturally variable conditions of a highly dynamic intertidal habitat that experiences regular upwelling events. After hypothesizing that these conditions may have led to local adaptation to low pH regimes, Evans and colleagues used their field measurements as well as future pH levels predicted for their study area [190] to inform the experimental pH conditions under which they examined the transcriptomic response of purple sea urchin larvae. In a polar habitat, Johnson and Hofmann [62] used observations made with autonomous pH sensors in McMurdo Sound, Antarctica [191] to select present-day and predicted future seasonal pH treatments. Applying these pH values, the authors compared shorter and longer-term acclimation to low pH levels in Antarctic pteropods by examining changes in gene expression [62]. In a study by Maas and colleagues [58], knowledge of their study organism’s behavior and natural environment was used to inform the experimental design. Laboratory CO_2_ conditions were manipulated to mimic levels that were experienced during diel vertical migrations of the thecosome pteropod *Clio pyramidata* to examine its short-term transcriptomic response of elevated CO_2_ [58]. Studies such as these underscore the functionality of understanding how organism ecology can vary between different marine systems and the importance of taking this into account to accurately assess transcriptomic responses to pH.

## Caveats and considerations

The use of transcriptomic studies in understanding molecular and physiological responses to stress has been fundamental in identifying key processes and pathways modulated under OA stress. However, the utility of transcriptomics still comes with caveats. In particular, very few transcriptomic studies are validated with proteomic or enzymatic activity data, although see [57, 83, 88]. There can be a disconnect between changes in gene expression and protein levels, especially in regard to time-scales of regulatory responses in the cell and when stress occurs during early development, which may favor different compensatory mechanisms to combat stress as to not disrupt developmental processes. In addition, it is possible that other mechanisms may modulate changes in phenotypes in response to stress, such as post-translational modifications, epigenetic processes and transposon activity. Regardless, the ease and relative cost-effectiveness of transcriptomics have allowed the organismal OA community to reveal broad consistent patterns across a wide variety of taxa (Table 1).

Two of the major caveats in comparing transcriptomic studies are defining gene function and assigning appropriate orthologs. In reality, interpreting functions of specific genes in many of the marine metazoans is based on strong, perhaps unfounded, underlying assumptions based on conserved gene function and orthology. There are exceptions to this, such as *S. purpuratus*, for which many gene annotations have been manually curated and functions during development described in depth [192, 193]. Therefore, functional understanding of gene expression patterns in many non-model systems is best carried out using broad functional groups (Gene Ontology Analysis) or by clustering groups of genes with similar patterns in a manner that is unbiased to gene function, such as Weighted Gene Co-Expression Network Analysis (WGCNA) [194], as suggested in a recent review [195]. This analysis is also recommended as it is evident that there are differences in bioinformatic pipelines used to identify candidate genes/genes of interest, as studies often choose to discuss candidate genes based on prior biases regarding their system of interest. While outside the scope of this review, there are other detailed reviews examining different bioinformatic methodologies to approach gene expression and transcriptomic data [195–198].

## Conclusions

In this review we have highlighted broad transcriptomic patterns expressed by marine metazoans in response to OA. The studies reviewed here have laid a strong foundation to further explore mechanisms of organismal responses to OA. Looking forward, it is critical to build from these studies to gain a stronger picture of how transcriptomics can inform the complexity of organism-environment interactions. Future work should focus on more mechanistic explanations through functional genetic studies that characterize gene function in an ecologically relevant context. In addition, transcriptomics should be more often compared with high throughput examinations of epigenetic marks, post-translational modifications, transposon activity, metabolomics and interactions with microbiomes/symbionts, all of which have the power to modulate phenotypes in concert with transcriptomic changes. The wealth of available transcriptomic data lends itself to comparative transcriptomics aimed to identify biomarkers of organismal health and stress, which could be used as tools in management and conservation of threatened ocean systems. Further, future studies should better integrate natural environmental fluctuations. Despite some restrictions and caveats of comparative transcriptomics, it remains a valuable approach to investigate pressing zoological issues such as OA, and for example, the emergence of marine heatwaves [199, 200]. Overall, comparative transcriptomics will continue to play a strong role in studying the response of marine metazoans to ocean change and opens up avenues of future research.

## Data Availability

The data presented in this manuscript are extracted from published literature.

## Abbreviations

CA: Carbonic anhydrase
CSR: Cellular stress response
EDS: Early developmental stage
FAD: Flavin adenine dinucleotide
GAPDH: Glyceraldehyde 3-phosphate dehydrogenase
HSP: Heat shock protein
OA: Ocean acidification
*p*CO_2_: Partial pressure of carbon dioxide
PNG: Papua New Guinea
qPCR: quantitative polymerase chain reaction
RNA-Seq: RNA sequencing
ROS: Reactive oxygen species
SEM: Scanning electron microscopy
TRAF: Tumor necrosis factor receptor associated factor
